# Comparative evaluation of first molar odontometry and mandibular measurements technique for gender determination

**DOI:** 10.6026/973206300222196

**Published:** 2026-04-30

**Authors:** Sanjeet Singh, Ruby Singh, Paramjit Singh, Kanika Sharma, Rumaisa Pandit, Priti Rawat

**Affiliations:** 1 Department of Oral Pathology & Microbiology, DJ College Of Dental Sciences & Research, Modi Nagar, Uttar Pradesh, India

**Keywords:** Forensic odontology, sexual dimorphism, diagonal odontometry, linear odontometry, orthopantomograms

## Abstract

Forensic odontology leverages durable teeth and mandibular structures to estimate sex in mass disasters and criminal investigations, yet optimal measurement approaches remain underexplored. Therefore, it is of interest to compare linear (mesiodistal, buccolingual) and diagonal (mesiobuccal-dentilingual, distobuccal-mesiolingual) odontometric dimensions of mandibular first molars and ramus morphometry on OPGs in 140 Indian adults (70 males, 70 females; 18-35 years). Males exhibited larger values across parameters; diagonal odontometry showed the highest dimorphism (9.93% ML-DB36) and accuracy (95% logistic regression) versus linear (80%, buccolingual best) and ramus measures (65.7% max, coronoid/condylar heights optimal). Diagonal measurements outperformed linear and ramus parameters, challenging traditional approaches to mandibular sex assessment. Thus, diagonal odontometry is superior (95% accuracy) for Indian populations, suggesting multimodal 3D validation for global applicability.

## Background:

Forensic odontology is critical for identifying a human being, especially when a mass disaster, natural calamity or criminal event occurs and traditional methods are not applicable [1]. Dental evidence is therefore considered one of the best personal identification aids. Dental identification has been officially recognized by the International Criminal Police Organization (INTERPOL) as a major technique in Disaster Victim Identification (DVI), whereby forensic significance has now been approved worldwide [2]. Of all craniofacial features, the mandible and more so, the ramus are highly sexually dimorphic because of the variation in the growth pattern, muscle attachment and functional remodeling between males and females [3]. The morphological alterations in the ramus are also age dependent and therefore, the dimension of the ramus is a good parameter in forensic sex determination [4]. The quantitative approach to analyzing tooth size, odontometry, has turned out to be a viable and economical technique for measuring sexual dimorphism and diagonal odontometric measurements, such as mesiobuccal/dentilingual (MBDL) and distobuccal/mesiolingual (DBMLE), which have been suggested as reliable alternatives [5]. The radiographic evaluation of mandibular ramus size, such as ramus breadth, condylar height and coronoid height, is another enhancement to forensic analysis, as it provides consistent and objective result [6]. Although the scientific evidence supporting the use of odontometric and mandibular morphometric techniques as independent procedures is increasing, comparative research using linear and diagonal measurements of first molar odontometry alongside mandibular ramus morphometry is scarce [7]. This can be an integrated solution that enhances reliability and applicability in the various forensic circumstances. Therefore, it is of interest to assess linear and diagonal odontometric data of a first molar to determine sexual dimorphism and to assess the morphometric parameters of the mandibular ramus using radiographs, to improve the accuracy of sex determination in forensic sciences.

## Materials and Methods:

In the current research, a total of 140 people (1835 years) were included and their Orthopantomographs (OPGs) and associated maxillary and mandibular dental casts were obtained. Inclusion and Exclusion Criteria Participants under 18 years of age, those 35 years of age and those with incomplete permanent dentition, severe physiological and pathological tooth wear and ambiguous OPGs were excluded. Patients who had missing, fractured, malformed or restored first molars; missing premolars; radiographic artifacts; mandibular lesions; a history of trauma; or TMJ abnormalities were eliminated. The maxillary and mandibular dental casts were measured by means of a digital vernier caliper (precision 0.01 mm). Mesio-distal (MD) and bucco-lingual (BL) values of first molars were measured and the diagonal measurements (mesio-buccal to disto-lingual and mesio-lingual to disto-buccal) were used at the height of contour and cervical line. A single examiner was used in all measurements. Digital measurements were performed using image analysis software and a mouse-driven method on 10 randomly selected casts after one week, with Kappa ranging from 0.81 to 0.90. The measures evaluated were the maximum and minimum ramus width, the maximum height of the ramus, the condylus and coronoid process, the gonial angle and the bigonial width. All measurements were performed digitally using a mouse-driven method.

[1] A: Maximum width of ramus: It is the largest anteroposterior diameter of the ramus.

[2] B: Ramus minimum width: This is the minimum anteroposterior diameter of the ramus.

[3] C: Maximum height of condylum: Starting with the highest point of the condylum of the mandible to the lowest point of the mandible.

[4] D: Maximum ramus height: This is the distance between the highest point of projection on the mandibular condylar projection and the lower border of the bone.

[5] E: The greatest distance between the bone and the lowest point to the coronoid: the projective distance between the coronoid and the lowest point of the bone. It is an oblique outline of what passes down at the lowest points of the gonial angle and the lower border of the mandibularbody to the posterior borders of the condyle and ramus.

[6] F: These two outlines created the gonial angle at their intersection point.

[7] G: Bigonial width: This is the horizontal distance between two goni, which is calculated between the right and left goni.

[8] The data analysis was done through SPSS version 20. A p-value below 0.05 was considered significant.

## Results:

A total of 140 subjects (70 males, 70 females) were assessed using mandibular ramus measurements from OPGs and odontometric measurements from dental casts for sexual dimorphism and sex-classification accuracy. Males showed higher mean ramus values than females, except for the gonial angle (slightly higher in females). Significant dimorphism was found for maximum coronoid, condylar and ramus heights (p<0.05), with the highest dimorphism in maximum coronoid height (19.67%) and maximum condylar height (18.23%). Table 1 (see PDF) identified minimum ramus width and gonial width as significant predictors (p<0.05). Accuracy: 65.7%. Males had larger MD and BL dimensions; significant differences were seen for MD16, MD26, BL16, BL26, BL36 and BL46 (p<0.05). BL showed greater dimorphism than MD, with the highest for BL26 (4.93%), BL46 (3.61%) and BL36 (0.85%). Predictors: MD46, BL36, BL46 (p<0.05). Accuracy: 80% (Table 2 - see PDF). Diagonal variables showed strong dimorphism, with males larger (p<0.001). The highest dimorphism was ML-DB36 (9.93%), CML-DB46 (5.79%) and CMB-DL16 (4.61%). Significant predictors included MBDL16, MLDB26, MLDB36 and CMLDB16 (p<0.05). Accuracy: 95% (Table 3 - see PDF). Table 4 (see PDF) shows that the diagonal odontometry performed best (95%), followed by linear (80%) and OPG (65.7%). Combining dental and mandibular metrics may further improve sex estimation.

## Discussion:

In the case of mass disaster situations where bodies are dismembered, burnt or rotting, forensic identification becomes extremely difficult to perform by using the traditional means of identifying an individual. Their resistance to environmental affronts and durability make teeth and mandibular structures key elements in gender determination, an essential first stage in identification. The current research relied on a sexual dimorphism assessment based on odontometric evaluation of mandibular first molar and mandibular ramus sizes from orthopantomograms (OPGs), contrasting the diagnostic efficacy of linear, diagonal and radiographic measures. Sexual dimorphism in dental size has a strong track record, with males having larger teeth than females due to genetic, hormonal and functional factors. The males indicated a substantially higher degree of mesiodistal (MD) and buccolingual (BL) measurements of mandibular first molars than females, with the latter showing greater discriminative ability. Such results are in line with previous research, in which Franco *et al.* [3], Bhagyashree *et al.* [4] and Viciano *et al.* [5] concluded that, compared to MD measures, BL dimensions are more genetically predetermined and not as much affected by attrition Rai & Anand (2007) [6^6^]. The present study indicated the greatest level of sexual dimorphism and prediction accuracy for diagonal odontometric measurements. Since diagonal dimensions combine both MD and BL components and cross the occlusal table, they provide a more detailed display of crown morphology. This is why they perform well compared to linear measurements, especially in teeth with moderate wear or asymmetry. The same has been noted by Tabasum *et al.* and Zorba *et al.* who have highlighted the strength of diagonal measurements in forensic sex estimation [7, 8]. An extreme level of sexual dimorphism in the peak coronoid height, condylar height and ramus height proves the hypothesis that sexual dimorphism in vertical mandibular growth is highly sex dependent. Pereira *et al.* [9] state that there is mass remodeling of the mandible due to muscle hypertrophy in males, which is mediated by testosterone and mainly on the ramus and condylar parts of the mandible. These results have been confirmed by Litha *et al.* [10] and Sairam *et al.* (2016) [11], who found that the most predictive variables for sex, using panoramic radiographs, are ramus height and condylar height. A larger gonial angle is observed in females and these findings are in agreement with those of Arthanari *et al.* (2024) [12] and Huumonen *et al.* (2010) [13], who noted reduced masseteric muscle activity and a shorter ramus. This morphological difference also confirms the application of the gonial angle as an additional parameter in sex determination. Significant sexual dimorphism could also be seen in mandibular ramus measurements made with OPGs, where males were taller on ramus, condylar and coronoid height, with females having a relatively larger gonial angle. These data are consistent with previous reports, which attributed these variations to skeletal development and the higher activity of the masticatory muscles in males [10, 14 and 15]. Nevertheless, even with statistically significant differences, the measurements using OPG showed moderate classification accuracy, likely because panoramic radiography has inherent limitations, including magnification errors, distortion and a two-dimensional representation. Nonetheless, not all mesiodistal dimensions were dimorphic, i.e., MD36. This is in line with the results of Zorba *et al.* (2011) [8], who postulated that MD dimensions are more prone to interproximal wear, dental crowding and population-specific differences and that their use is less reliable for estimating sex. Comparison of the three approaches showed that the diagonal odontometric measurements had the highest predictive accuracy, followed by linear odontometric measurements and OPG-derived mandibular parameters. Such a hierarchy underscores the superiority of direct dental measures over radiographic techniques. It supports the use of diagonal odontometry as a valid means of determining gender in any forensic context. Notably, the use of dental and mandibular parameters further increased overall accuracy, indicating the advantage of a multifactorial approach, particularly when complete or damaged remains are involved. Variability in the population is also a factor considered in forensic odontology. The results of the current research provide population-related reference data that support the idea that localized standards are necessary to enhance the accuracy and applicability of sex estimation models in odontometry.

## Conclusion:

There is significant sexual dimorphism in the sex estimation of mandibular and dental measurements. Data shows that diagonal odontometry is the best method for estimating gender in forensic odontology. This is similar to the approach that uses dental casts and OPG measurements.
